# Chronic gestational hypoxia accelerates ovarian aging and lowers ovarian reserve in next-generation adult rats

**DOI:** 10.1096/fj.201802772R

**Published:** 2019-03-19

**Authors:** Catherine E. Aiken, Jane L. Tarry-Adkins, Ana-Mishel Spiroski, Anna M. Nuzzo, Thomas J. Ashmore, Alessandro Rolfo, Megan J. Sutherland, Emily J. Camm, Dino A. Giussani, Susan E. Ozanne

**Affiliations:** * University of Cambridge Metabolic Research Laboratories and Medical Research Council (MRC) Metabolic Diseases Unit, Wellcome Trust–MRC Institute of Metabolic Science, Addenbrooke’s Treatment Centre, Addenbrooke’s Hospital, Cambridge, United Kingdom;; † Department of Obstetrics and Gynaecology, University of Cambridge, Cambridge, United Kingdom;; ‡ Department of Physiology, Development, and Neuroscience, University of Cambridge, Cambridge, United Kingdom;; § Dipartimento di Scienze Chirurgiche, Universita degli Studi di Torino, Turin, Italy

**Keywords:** ovary, follicles, reproductive aging, fetal hypoxia, developmental programming

## Abstract

Chronic fetal hypoxia is a common complication observed in human pregnancy, impacting pregnancies across global contexts. Exposure to chronic intrauterine hypoxia has major short- and long-term consequences for offspring health. However, the impact of chronic gestational hypoxia on female reproductive system development is unknown. We aimed to understand the impact of exposure to chronic fetal hypoxia on the developing female reproductive system. Wistar rat dams underwent normoxia (21%) or hypoxia (13%) during pregnancy. Postnatally, all female offspring were maintained in normoxic conditions into early adulthood. Female rats exposed to chronic gestational hypoxia (13%) during their intrauterine development had decreased ovarian primordial follicular reserve compared to controls (*P* < 0.05). Adult females who had been exposed to chronic fetal hypoxia had significantly reduced somatic ovarian telomere length (*P* < 0.05) and reduced ovarian protein expression of KU70, a critical component of the DNA-activated protein kinase repair complex (*P* < 0.01). Gene expression of NADPH oxidase 2–mediated oxidative stress markers was increased (*P* < 0.05). Exposure to chronic hypoxia during fetal development leads to accelerated aging of the somatic ovary and decreased ovarian reserve in adulthood. Ovarian aging is highly sensitive to gestational hypoxia, with implications for future fertility in next-generation offspring of high-risk pregnancies.—Aiken, C. E., Tarry-Adkins, J. L., Spiroski, A.-M., Nuzzo, A. M., Ashmore, T. J., Rolfo, A., Sutherland, M. J., Camm, E. J., Giussani, D. A., Ozanne, S. E. Chronic gestational hypoxia accelerates ovarian aging and lowers ovarian reserve in next-generation adult rats.

Chronic gestational hypoxia is a common feature of a number of suboptimal intrauterine environments, including placental insufficiency, preeclampsia, maternal smoking, and pregnancy at high altitude (1, 2). Exposure to chronic hypoxia during gestation adversely influenced fetal and placental development and is associated with adverse pregnancy outcomes (2–6). The short-term adverse effects of chronic gestational hypoxia include increased risks of late miscarriage, fetal growth restriction, and low birth weight (3–8). Chronic gestational hypoxia also has long-term effects on the physiology of exposed offspring, termed developmental programming. The effects of gestational hypoxia are best characterized in the cardiovascular system, where the impact of low oxygen tension on the developing heart and vasculature has been extensively studied in animal models (1, 3). The consequences of a suboptimal fetal environment on long-term reproductive health is an underexplored area in the field of developmental programming but an area of huge importance given that the reproductive system is the mediator of information across generations. In particular, the impact of chronic hypoxia on the development of the female reproductive system is unknown.

The developing female reproductive system is particularly vulnerable to the impact of a suboptimal intrauterine environment because of the specific developmental windows during which ovarian reserve is established. Ovarian reserve refers to the total finite number of primordial follicles remaining in both ovaries at any point in life and is the key determinant of fertility potential in the female (4). Disruptions to the fetal environment during the crucial phase of ovarian follicular endowment result in a decreased ovarian reserve in adult reproductive life (5–9). *In vitro* evidence suggests that the ovarian follicle is particularly sensitive to oxygen tension. Oocyte development within follicles in the adult ovary is markedly influenced by the oxygen content of the follicular fluid (10), with hypoxic follicles containing a higher percentage of oocytes with derangements of chromosomal organization. Therefore, there is a strong rationale to hypothesize that follicular dynamics in the developing follicles in the ovary *in utero* may be highly influenced by exposure to chronic gestational hypoxia. In this study, we investigated whether ovarian reserve in the young adult female is influenced by exposure to chronic hypoxia during gestation and determined underlying mechanisms.

## MATERIALS AND METHODS

All animal experiments were approved by the University of Cambridge Animal Welfare and Ethical Review Board. All animal experiments were conducted in accordance with the British Animals (Scientific Procedures) Act (1986) and were compliant with European Union Directive 2010/63/EU.

### Study design

Wistar rat dams at 10–12 wk of age (Charles River, Margate, United Kingdom) were housed in individually ventilated cages (21% oxygen, 70–80 air changes/h) under standard conditions. All animals were fed a standard laboratory chow diet (20% protein; Arie Blok Animal Nutrition, Woerden, The Netherlands) and fed *ad libitum* with free access to water. After initial acclimatization (10 d), they were mated with fertile male Wistar rats (*n* = 14), and pregnancy was confirmed through the observation of a vaginal plug. The day of the plug was designated d 0 of pregnancy (full term 21–22 d). Upon confirmation of pregnancy, dams were weighed and housed individually. On d 6 of pregnancy, dams were randomly divided into 2 groups: control (21%) and hypoxic (13%) pregnancy (*n* = 8 per group). Pregnant rats assigned to the hypoxia group were placed inside a chamber, which combined a PVC isolator with a nitrogen generator, as previously described in refs. 11 and 12. Hypoxic pregnancies were maintained at a constant inspired fraction of oxygen of 13% from d 6 to 20 of gestation. This model of hypoxic pregnancy does not decrease maternal food intake (11). All dams were delivered under normoxic conditions. There were no complete pregnancy losses in either group during the study. The respective litter sizes were 12.3 ± 1.0 pups in the normoxia group compared to 9.3 ± 1.2 pups in the hypoxia group (*P* < 0.05). Gestational length averaged 20 ± 1 d in both normoxic and hypoxic groups. Normoxia (21%) was maintained for all animals during lactation, weaning, and thereafter. Following determination of birth weight, litters were culled to 4 males and 4 females to standardize nutritional access and maternal care during suckling (11, 12). All pups were suckled by their own mothers. At 4 mo of age, adult female pups underwent euthansia. At *postmortem*, the reproductive tract tissues were harvested and weighed fresh, immediately after dissection. One ovary from each animal was snap-frozen in liquid nitrogen and the other fixed in formalin-paraldehyde. The fixed ovaries were sectioned and subjected to hematoxylin and eosin (H&E). An equal distribution of estrous cycle stages in each group was confirmed using the serial sections of a whole H&E-stained ovary prepared for primordial follicle counting. However, the study was not powered for comparisons between estrous cycle stages; thus, parameters were selected to be nonvarying with cycle stage. Sample analysis was performed using project codes to blind the investigators to the experimental groups. The adequacy of the sample size was determined *via* a power calculation based on the effect sizes for ovarian primordial follicle counts in Wistar rats reported in our previous studies (6, 13) using an α level of 0.05 to give power of 0.8.

### Primordial follicle counts

Primordial follicle counts were performed as we previously described (6, 13). Fixed ovaries were processed for microscopy and the entire ovary sectioned at 8 μm. Every ninth section was stained with H&E for morphometric analysis (72 μm between analyzed sections). Only follicles with a visible oocyte nucleus were counted in order to avoid repeat counts of the same follicle (14). Primordial follicles were identified morphologically by the presence of a single layer of flattened granulosa cells surrounding the oocyte (15) (Supplemental Fig. S1). The total volume of each ovary was derived (section areas × section thickness × number of sections), and the follicle count normalized to ovarian mass as follicles per cubic millimeter of ovarian tissue.

### Telomere length analysis

High-MW DNA was extracted using the DNeasy Blood and Tissue Kit (Qiagen, Germantown, MD, USA) according to the manufacturer’s instructions. DNA quantity and purity was determined using a Nanodrop spectrophotometer (Thermo Fisher Scientific, Waltham, MA, USA). Agarose gels were run to ensure all DNA samples were of high MW. DNA (1.2 μg) was digested with *Hinf*I and *Rsa*I restriction enzymes for 2 h at 37°C. The restricted samples were quenched with 5 times SDS loading buffer (Roche, Basel, Switzerland) and loaded onto agarose gels containing SYBR safe stain (Thermo Fisher Scientific). After pulsed-field gel electrophoresis, the gels were checked for nonspecific degradation of an undigested DNA control and complete digestion of the enzyme-restricted DNA by visualizing the stained gels under UV light (Syngene, Bangalore, India). The separated DNA fragments were transferred to nylon membrane (Roche) by Southern blotting, and telomeric repeat length was determined using a commercial method of chemiluminescent detection as previously described by Tarry-Adkins *et al.* (16). MW markers on each gel were a midrange pulsed-field gel marker (New England Biolabs, Ipswich, MA, USA) and dioxygenin (low range) molecular-weight marker (Roche). Standard digested genomic samples of DNA from a 4-mo control animal were also included on each gel to verify digestion efficiency. Telomere signals were analyzed using Adobe Photoshop (Adobe, San Jose, CA, USA) and Alpha-Ease software (Alpha Innotech, San Leandro, CA, USA). Telomere length was measured as previously described by Tarry-Adkins *et al.* (16).

### Gene expression analysis

An initial panel of 32 candidate genes was developed to test which molecular pathways might be altered in the somatic ovary following exposure to chronic gestational hypoxia. These genes were chosen based on *1*) previous work on the effects of developmental programming on ovarian, *para*-ovarian adipose tissue, and oviductal gene expression (5, 17, 18); *2*) knowledge of programming mechanisms in other organ systems in the same gestational hypoxia rat model (11, 19, 20); and *3*) relevant literature review. RNA was extracted from snap-frozen ovaries using a miRNeasy Mini Kit (Qiagen) following manufacturers’ instructions, with the addition of a DNaseI digestion step to ensure no genomic DNA contamination. RNA quantification was performed using a NanoDrop spectrophotometer (Nanodrop Technologies). For the RT-PCR process, RNA (1 μg) was reverse transcribed to cDNA using oligo-dT primers and Moloney murine leukaemia virus (M-MLV) V reverse transcriptase (Promega, Madison, WI, USA). Gene expression was determined using custom designed primers that were designed within 1 exon (**Table 1**; MilliporeSigma, Burlington, MA, USA) and SYBR Green reagents (Thermo Fisher Scientific) as previously described by Tarry-Adkins *et al.* (21). Quantification of gene expression was performed using a Step One Plus RT-PCR machine (Thermo Fisher Scientific). All cDNA samples were run against a genomic DNA standard curve of known concentrations using a commercially available rat genomic DNA standard (MilliporeSigma) in order to express gene expression as average copy number. Equal efficiency of the reverse transcription of RNA from all groups was confirmed through quantification of expression of the housekeeping gene cyclophilin A (*ppia*), the expression of which did not differ between groups.

**TABLE 1:** Primer sequences and product size

	Primer sequence, 5′–3′		
Primer	Forward	Reverse	Product size (bp)
*Ogg1*	CTTAATGGCCCTGGACAAAC	CGTAGTCACGATGGGCAAT	74
*Nth1*	GATTTTGCCTTCCTGTCCATC	GAAGCCCAAAACCCTCAGA	79
*Neil1*	CAGAAGGTCAAGGCCAAACT	CTCACCCAGCTGAACCACTT	84
*Xrcc1*	GGGGAGAAGGCACAGAGC	TCTGGAAGCCACCTGAGCAC	96
*Ku70*	ACTGAGGGACATCTGCAAGG	ACTGAGGGACATCTGCAAGG	84
*Ku80*	GACATGAAGCTCTGGCCATC	TGTCTGTAGGGACCTGGAGTG	68
*Pot1*	CTGCAACTAAAGCGCCAGAC	CGGTGGTCCAGATCTTTGAT	69
*Chk1*	TTGGTCAAAAGGATGACACG	GGTCTCTTTCAGGCATTGGT	72
*Chk2*	GAGACCTCCCTGATGAGGAC	AGCAGTCCCGTTGGAGATAA	78
*Cdk4*	GATTGCCTCCAGAAGACGAC	ATCTCCGGCACCACTGACT	100
*Cdk6*	GCCCTGAATCACCCGTACT	GCTGGACGACAGGTGAGAAT	72
*P53*	CCTATCCGGTCAGTTGTTGG	CGTATGAGGGCCCAAGATAG	89
*P21*	TGCAAGAGAAAGCCCTGAAG	TGAATGAAGGCTAAGGCAGAA	96
*P16ink*	GCTCGACCTGGCCCTAGA	GCGGAGGAGAGTAGATACCG	69
*Hif1α*	TCAAAAGCAGTGACGAAGGA	TGGGTAGAAGGTGGAGATGC	68
*Nfκβ*	CTTCTCGGAGTCCCTCACTG	TAGGTCCATCCTGCCCATAA	80
*Xo*	GAGAAGGTCTCCAGCAGTGG	GCATGCGGAAATCTGGATA	86
*Gp91^phox^*	CGAAGCCTTGGCTAAAACTCT	TCCTTGTTGAAGATGAAGTGGA	87
*P22^phox^*	GTGAGCAGTGGACTCCCATT	GTAGGTGGCTGCTTGATGGT	76
*P67phox*	CCGATAACCGGACAACAGAG	CAGGTCTTCTGGCTGGGTAG	72
*Nrf2*	AGCAAGACTTGGGCCACTTA	GATGGAGGTTTCTGTCGTTTTC	78
*Hmox1*	TAACCAGGATCTCCCCAAGA	TTAGAGTGCTGTGGCAGGTG	71
*Gpx1*	CACCCGCTCTTTACCTTCCT	CGGGGACCAAATGATGTACT	75
*Mnsod*	TGACTATGTAATGTTTTATCAGTTGGA	GTTGCTGACCACAGCCTTTT	91
*CuZnsod*	TTGTGGTGTGATTGGGATTG	CAGTTTAGCAGGACAGCAGATG	80
*Ecsod*	ATCCCATAAGCCCCTAGCAT	ATTCGACCTCTGGGGGTAAG	84
*Catalase*	TTGGATCATGTCTTCCAAAAA	GGGAAAAGGAATCCGATCAA	83
*Alox12*	TGTGCTCAGCCAATTTCAAG	GGTATTCGTAGGGCAGGTCA	89
*Alox15*	GCTGTGCTGAAGAAGTTCAGAG	GCCGCAGGTACTCATAAGGT	85
*Ppia*	TGAGAACTTCATCCTGAAGCATACA	CATTTGTGTTTGGTCCAGCATT	89

*Neil1*, endonuclease VIII–like 1; *Xrcc1*, X-ray repair cross complementing 1.

### Protein quantification

Protein was extracted from whole-tissue lysates of snap-frozen ovaries, as previously described in refs. 18 and 22. To ensure equal protein loading, protein assays were performed on all samples to ensure that each sample was diluted to the same protein concentration (1 mg). Protein (20 μg) was loaded onto 10, 12, or 15% polyacrylamide gels, dependent upon the MW of the protein to be measured. The samples were electrophoresed and transferred to polyvinylidene fluoride membranes. Detection steps used the following primary antibodies; 8-oxoguanine DNA glycosylase (OGG1; NB100-106, 1:1000; Novus Biologicals, Centennial, CO, USA), NTH1 (11154-1-AP, 1:1000; Proteintech, Rosemont, IL, USA), hypoxia-inducible factor 1α (HIF1α; Ab51608, 1:1000; Abcam, Cambridge, MA, USA), catalase (Ab1877-10, 1:10,000; Abcam), MnSOD (06–984; Upstate, Watford, United Kingdom), copper/zinc superoxide dismutase (CuZnSOD; 10269-1-AP, 1:1000; Proteintech), NADPH oxidase 1 (GP91^phox^; 19013-1-AP; Proteintech), neutrophil cytosolic factor 2 (P67^phox^; 15551-1-AP, 1:1000; Proteintech), xanthine oxidase (XO; SC-20991, 1:200; Santa Cruz Biotechnology, Dallas, TX, USA), tumor protein 53 (P53; MAB1355, 1:1000; R&D Systems, Minneapolis, MN, USA), cyclin-dependent kinase inhibitor 2A (P16^ink^; Ab189034, 1:1000; Abcam), KU70 (10723-1-AP, 1:1000; Proteintech), KU80 (NB100-508, 1:1000; Novus Biologicals). Anti-rabbit secondary antibodies (1:2000; Cell Signaling Technology, Danvers, MA, USA) were utilized for all primary antibodies except P53, which required an anti-mouse secondary antibody (1:2000; Cell Signaling Technology) (Supplemental Fig. S2). Equal protein loading was confirmed by staining electrophoresed gels with Coomassie Blue (Bio-Rad, Hercules, CA, USA) to visualize total protein. This methodology was selected to avoid the use of housekeeping proteins that may be vulnerable to expression differences following developmental hypoxia exposure (23, 24). To ensure that the chemiluminescent signal changed in a linear manner, the ratio between loading controls (100 and 50% pooled sample) was confirmed for each detected protein.

### Statistical analysis

Maternal hypoxia effects were compared between groups using 2-tailed Student’s *t* tests. In order to correct for multiple hypothesis testing of gene expression levels, *P* values were transformed to *q* values to take account of the false discovery rates using the p.adjust function in the R statistical package (R Foundation for Statistical Computing, Vienna, Austria). This adjustment was designed for this study in order to take account of the specific number of genes that were tested within the initial screen (25) and therefore to ensure that the *P* values were optimally transformed. Data are represented as means ± sem. Where *P* values are reported, an α level <0.05 was considered statistically significant. All data analysis was conducted using the R statistical software package v.2.14.1 (R Foundation for Statistical Computing). Only ovaries of 1 female offspring per litter were used for analysis to account for within litter variation. Therefore, in all cases, *n* refers to the number of litters, and *n* = 8 was used for all groups.

## RESULTS

There was no significant difference in the body weight of female rats at 4 mo of age exposed to gestational hypoxia compared to those that experienced normoxia; however, there was a trend toward a slightly lower body weight in the hypoxia group (*P* = 0.06; **Table 2**). Ovarian weight was not significantly different between the groups, whether expressed as absolute organ weight or normalized to body weight (Table 2).

**TABLE 2:** Body weight and ovary weights in adult female rats exposed to gestational hypoxia and normoxia

Parameter	Experimental group		*P* value
	Normoxia	Hypoxia	
Body weight (g)	313.1 ± 5.9	297.5 ± 5.9	0.06
Ovarian weight (g)	0.08 ± 0.01	0.07 ± 0.01	0.18
Ovarian weight: body weight ratio	0.02 ± 0.00	0.02 ± 0.00	0.49

**Figure 1:**
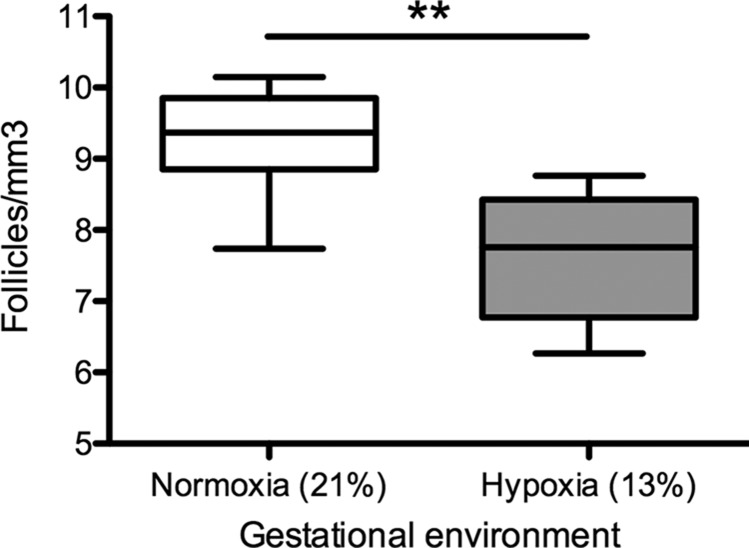
Primordial follicular reserve in adult female rats exposed to gestational hypoxia compared to normoxia. Box plots: median ± upper and lower quartiles; whiskers: maximum and minimum values. Open bars: normoxia (21% oxygen) during gestation; gray bars: hypoxia (13% oxygen) during gestation. Primordial follicle count is shown normalized to cubic millimeters of ovarian tissue. **P < 0.01.

Primordial follicle counts per cubic millimeter of ovarian tissue at 4 mo of age were significantly lower in the gestational hypoxia than in the gestational normoxia group (*P* < 0.01; **Fig. 1**). Absolute follicle counts were also lower in the gestational hypoxia group than the normoxic group (131.0 ± 12.4 *vs.*183.7 ± 20.6 follicles per ovary, *P* < 0.05).

**Figure 2:**
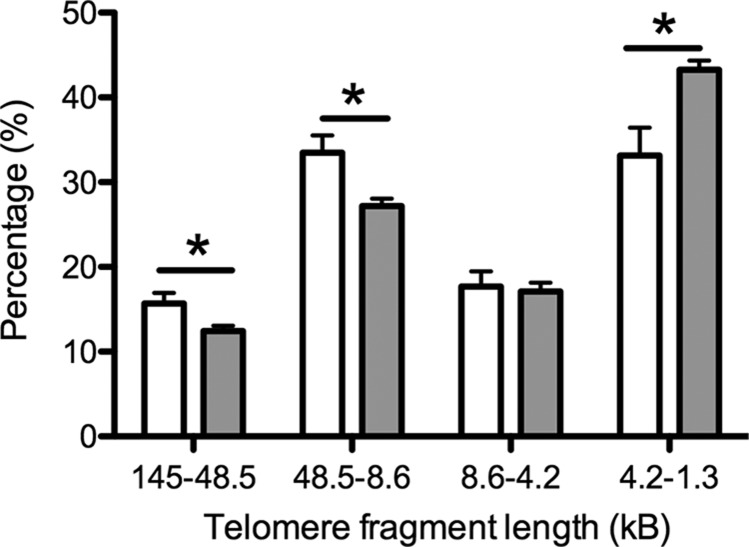
Ovarian telomere length in adult female rats exposed to gestational hypoxia compared to normoxia. Data shown as means ± sem. Open bars: normoxia (21% oxygen) during gestation; gray bars: hypoxia (13% oxygen) during gestation. *P < 0.05.

At 4 mo of age, there were significantly fewer very long (145–48.5 kB, *P* < 0.05) and long (48.5–8.6 kB, *P* < 0.05) telomeres in the somatic ovarian tissue of gestational hypoxia–exposed animals compared to the normoxic group (**Fig. 2**). Conversely, there was a higher proportion of very short telomeres in the hypoxia exposed group animals (1.1–4.2 kB, *P* < 0.05), strongly suggesting that telomere length maintenance is impaired in the somatic ovary following the developmental challenge of hypoxia *in utero*.

**Figure 3:**
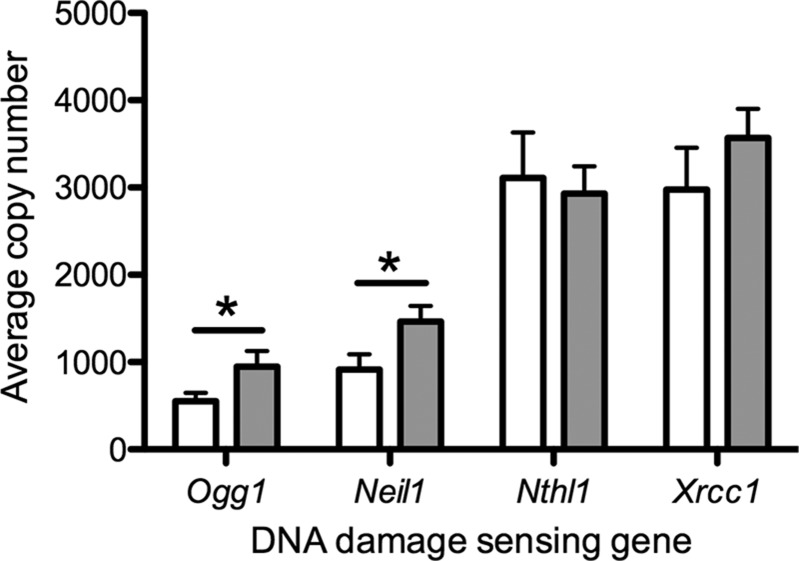
Effect of gestational hypoxia compared to normoxia on DNA damage–sensing gene expression in the ovary of adult female rats. Data shown as means ± sem. Open bars: normoxia (21% oxygen) during gestation; gray bars: hypoxia (13% oxygen) during gestation. *P < 0.05.

One possible mechanism of accelerated telomere shortening is impaired recognition of DNA damage. Accordingly, we measured the gene expression levels of a range of DNA–damage sensing and repair proteins in the hypoxia-exposed animals. Expression of *Ogg1* (*P* < 0.05) and endonuclease VIII–like 1 (*P* < 0.05) was elevated in the hypoxia-exposed group compared to the normoxic group (**Fig. 3**), which is in keeping with an increased burden of DNA damage in the hypoxia-exposed group. There was no difference in gene expression of either Nth-like DNA glycosylase 1 or X-ray repair cross complementing 1 in either group (Fig. 3). At the protein level, NTH1 was increased in the hypoxia-exposed group compared to the controls (*P* < 0.01), but there was no difference in the protein level of OGG1 (**Table 3**).

**TABLE 3:** Effect of gestational hypoxia compared to normoxia on protein expression in the ovary of adult female rats

Protein	Expression level		Significance level
	Normoxia	Hypoxia	
OGG1	100 ± 15	116 ± 24	NS
NTH1	100 ± 6	128 ± 5	<0.01**
GP91^phox^	100 ± 9	125 ± 14	0.08
XO	100 ± 10	122 ± 13	NS
HIF1α	100 ± 4	97 ± 7	NS
P67^phox^	100 ± 13	120 ± 9	NS
MnSOD	100 ± 5	82 ± 9	NS
CuZnDOS	100 ± 17	72 ± 6	NS
CATALASE	100 ± 4	79 ± 4	<0.05*

All reported *P* values have been adjusted to take account of multiple hypothesis testing. NS, not significant. *P < 0.05, **P < 0.01.

**Figure 4:**
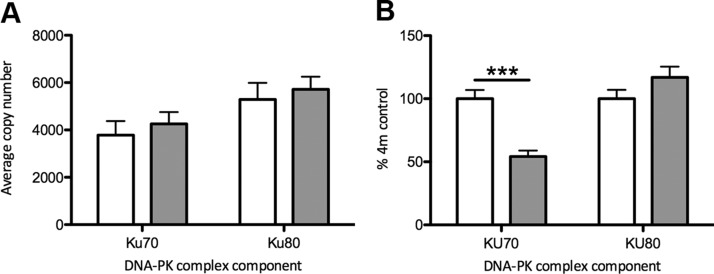
Effect of gestational hypoxia compared to normoxia on expression of components of the DNA-PK in the ovary. Data shown as means ± sem. Open bars normoxia (21% oxygen) during gestation; gray bars: hypoxia (13% oxygen) during gestation. *A*) Gene expression. *B*) Protein expression. Protein expression is represented as the percentage of the 4-mo normoxia group (assigned baseline value of 100%). ***P < 0.001.

Gene expression levels of the key functional subunit components of DNA-activated protein kinase (DNA-PK), *Ku70* and *Ku80*, primarily responsible for repairing double-stranded DNA breaks and hence playing a role in maintaining telomere length, did not vary significantly between the hypoxia- and normoxia-exposed groups (**Fig. 4*A***). However, at the protein level, there was a highly significant reduction in KU70 in the animals exposed to gestational hypoxia (*P* < 0.001), with no difference between groups in KU80 levels (Fig. 4*B*). Inability to repair double-stranded DNA breaks, despite adequate detection, is consistent with the accelerated telomere shortening observed in the somatic ovarian tissue of the group exposed to chronic gestational hypoxia. There were no differences between the hypoxic and normoxic groups in the gene expression of any other DNA damage–sensing or protection mechanisms that were assayed [protection of telomeres protein 1 (*Pot1*), checkpoint kinase 1 (*Chk1*), checkpoint kinase 2 (*Chk2*), *Cdk4*, *Cdk6*; **Table 4**].

**TABLE 4:** Effect of gestational hypoxia compared to normoxia on gene expression in the ovary of adult female rats

Gene	Expression level		Significance level
	Normoxia	Hypoxia	
*Pot1*	508 ± 91	382 ± 37	NS
*Chk1*	269 ± 62	222 ± 36	NS
*Chk2*	713 ± 94	660 ± 53	NS
*Cdk4*	7928 ± 2430	9761 ± 1035	NS
*Cdk6*	382 ± 76	354 ± 42	NS
*P53*	10,796 ± 2216	18,816 ± 1775	0.01**
*P21*	1892 ± 350	4367 ± 1086	0.08
*P16ink*	344 ± 79	592 ± 138	0.09
*Hif1a*	7267 ± 3940	23,130 ± 4682	0.02*
*Nfkβ*	7288 ± 1181	6429 ± 776	NS
*Nox2*	7716 ± 2815	10,145 ± 2142	NS
*Xo*	9012 ± 1970	5981 ± 856	NS
*Gp91^phox^*	10,489 ± 1853	15,066 ± 1715	0.05*
*P22^phox^*	356 ± 66	892 ± 199	0.03*
*P47phox*	1440 ± 224	1592 ± 221	NS
*P67phox*	134 ± 23	169 ± 43	NS
*Nrf2*	20,489 ± 3662	21,479 ± 1582	NS
*Hmox1*	2273 ± 284	2311 ± 222	NS
*Gpx1*	77,136 ± 14,644	82,485 ± 15,296	NS
*Mnsod*	11,513 ± 2248	12,716 ± 2207	NS
*CuZnsod*	31,951 ± 6327	89,260 ± 12,321	<0.001***
*Ecsod*	31,328 ± 5823	38,331 ± 3371	NS
*Catalase*	8898 ± 1169	8448 ± 1681	NS
*Alox12*	196 ± 40	303 ± 48	NS
*Alox15*	605 ± 100	479 ± 93	NS
*Ppia*	30,565 ± 5526	32,628 ± 4212	NS

All reported *P* values have been adjusted to take account of multiple hypothesis testing. *Nfkβ*, nuclear factor κ-β DNA binding subunit; *Nox2*, NADPH oxidase 2; NS, not significant; *P47phox*, neutrophil cytosolic factor 1. *P < 0.05, **P < 0.01, ***P < 0.001.

**Figure 5:**
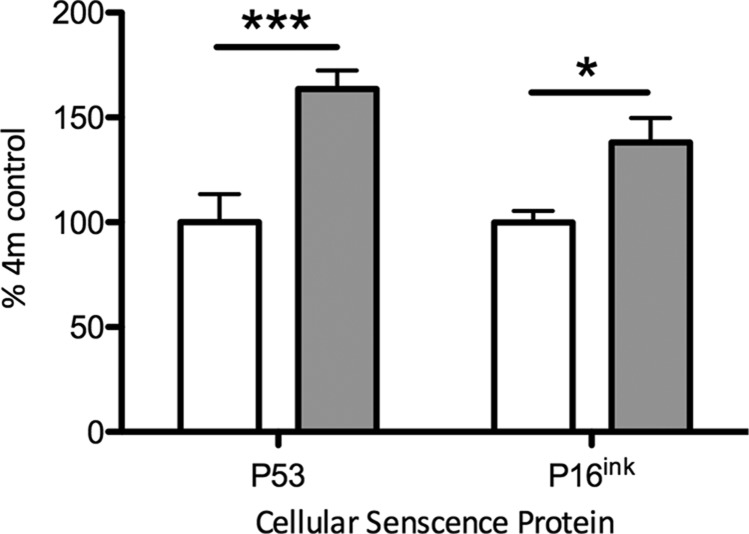
Effect of gestational hypoxia compared to normoxia on expression of cellular senescence proteins in the ovary. Data shown as means ± sem. Data are represented as the percentage of the 4-mo normoxia group (assigned baseline value of 100%). Open bars: normoxia (21% oxygen) during gestation; gray bars: hypoxia (13% oxygen) during gestation. P53, left; P16^ink^, right. ***P < 0.001, *P < 0.05.

Gene expression levels of *p53* were significantly higher in the somatic ovarian tissue of gestational hypoxia–exposed animals, than in normoxic controls (*P* < 0.01; Table 4). There were trends toward a similar increase in levels of cyclin-dependent kinase inhibitor 1 (*p21*) and *p16ink*, but these were not significant after correction for multiple hypothesis testing (Table 4). At the protein level there was a significant increase in both P53 (*P* < 0.001) and P16^ink^ (*P* < 0.05) in the hypoxia-exposed group compared to the normoxia-exposed group (**Fig. 5**).

There was a significantly higher gene expression of *Hif1α* in the somatic ovary following exposure to chronic gestational hypoxia than in the normoxic control group (*P* < 0.05, Table 4); however, there was no difference in expression levels of nuclear factor κ-β DNA binding subunit (Table 4). Various oxidative stress markers were included in the initial gene expression screen (Table 3). There was a specific up-regulation of oxidative stress markers *Gp91^phox^* (*P* < 0.05) and human neutrophil cytochrome b light chain (*P22^phox^*; *P* < 0.05) in the hypoxia-exposed group at the gene expression level, but other markers [*Xo,* neutrophil cytosolic factor 1*, P67^phox^*, nuclear respiratory factor 2 (*Nrf2*), heme oxygenase 1 (*Hmox1*), glutathione peroxidase 1 (*Gpx1*)] were not significantly different between experimental groups (Table 4). At the protein level, there was an increase in GP91^phox^ expression in the hypoxia-exposed group, but this did not reach statistical significance (*P* = 0.08; Table 4). There was no difference between groups at the protein level in expression of XO, P67^phox^, or HIF1α (Table 3).

In keeping with increased levels of oxidative stress in the somatic ovary in the hypoxia-exposed group, there was also a significantly higher gene expression of the cytoplasmic anti-oxidant *CuZnsod* (*P* < 0.001, Table 4). There was no difference in the gene expression levels of several other antioxidants included in the initial screen [*Mnsod*, extracellular superoxide dismutase (*Ecsod*), *Catalase*; Table 4] between the gestational hypoxia- and normoxia-exposed groups. There were no differences in antioxidant protein expression between the hypoxia- and normoxia-exposed groups, except for catalase, which was decreased in the hypoxia-exposed group (*P* < 0.05; Table 3). Gene expression levels of markers of lipid peroxidation included in the initial screen [arachidonate 12-lipoxygenase (*Alox12*), arachidonate 15-lipoxygenase (*Alox15*); Table 4] were unchanged between the gestational hypoxia- and normoxia-exposed groups.

## DISCUSSION

Hypoxia during fetal life is the final common pathway of a number of important pregnancy complications (1–3). Chronic gestational hypoxia may arise from maternal hypoxemia (for example, during pregnancy at high altitude) (26), maternal smoking (27), or insufficiency of utero-placental blood flow (28). A hypoxic intrauterine environment may also result from failure of conversion of the spiral arteries during early placental development (29). Failure to adapt the uterine blood flow to the demands of pregnancy also promotes chronic fetal hypoxia, which is associated with pregnancy complications including pregnancy loss, fetal growth restriction, and increased risk of preeclampsia (30, 31). Taken together, the various etiologies leading to chronic gestational hypoxia affect a large number of human pregnancies globally (3), including 389 million people who live at altitudes >1500 m and at least 70 million who live above 2500 m (25, 32). The immediate adverse impacts of chronic gestational hypoxia, including the high risk of fetal loss (33) and low birth weight (34), are well established. Many aspects of longer-term health in survivors of a hypoxic intrauterine environment, including adverse cardiovascular (11) and metabolic (12) impacts have also been characterized. However, our study provides a conceptual advance presenting important new evidence of a significant impact on reproductive potential through accelerated cellular aging in adult female offspring of hypoxic pregnancy.

We show that chronic gestational hypoxia leads to decreased ovarian reserve in female offspring in early adulthood. Ovarian reserve is a key determinant of female fecundity and hence a reduction in the number of primordial follicles available in early adulthood is highly likely to be associated with an early decline in fertility (35). Fecundity in later life relates to both oocyte quality and quantity; however, there is, as yet, no well-established method of reliably predicting oocyte quality (36). Hence, there is a reliance on occyte quantity. Our results suggest that accelerated aging in the somatic ovary in response to early-life hypoxia may be the result of a posttranscriptional reduction in expression of a component of the DNA-PK complex, which in turn prevents telomere maintenance and leaves ovarian follicular cells vulnerable to accumulating age-associated damage. It is thus highly plausible that accelerated reproductive aging is a key mechanism by which ovarian reserve in the next generation female offspring is reduced following exposure to chronic gestational hypoxia. Understanding the developmental basis of accelerated reproductive aging is particularly important in light of global trends toward increasing maternal age.

Physiologic early embryonic and fetal development proceeds in a low oxygen tension environment, with high levels of antioxidants, in order to protect the conceptus from potential oxidative damage during organogenesis (37, 38). However, after the establishment of the placental circulation, at the end of the first trimester in human pregnancy, oxygen tension rises dramatically (39). Failure of the oxygen tension to rise sufficiently during this developmental phase, whether as a result of limited utero-placental flow (40) or, as in our model and at altitude, low ambient oxygen levels, results in an increase in placental oxidative stress (41, 42). This early accumulation of oxidative stress has important consequences for the development of the fetal heart (43) and potentially other organ systems (3, 43, 44). Of particular note, endowment of the ovarian follicular reserve occurs concomitantly with the physiologic rise in oxygen tension at around 12 wk in human pregnancy (39). In the fetal ovary at this stage, the primordial germ cells have completed migration to the genital ridge and enter meiosis, irrevocably setting the maximum potential number of oocytes and commencing the oxygen-sensitive process of follicular development (45). Hence, there is rationale to consider whether the decreased ovarian reserve that we observe in adulthood may be a consequence of failure to experience the expected increase in oxygen tension during this crucial period of early development.

In keeping with the findings of this study, previous work has demonstrated a similar phenotype of early renal aging in response to developmental hypoxia (46). However, previous work exploring the developmental response to hypoxia in the developing cardiovascular system does not show a direct accelerated aging effect (47). Thus, the long-term impacts of developmental hypoxia on the female reproductive tract are likely to represent a tissue-specific effect rather than a ubiquitous response to a developmental stressor.

Numerous studies in human populations (35, 48) have established the link between reduced ovarian reserve and female reproductive potential. Evidence from >15,000 healthy women across cultural contexts in Latin America suggests that high altitude (>2000 m, hypobaric hypoxia) is associated with earlier age at menopause (49). Smaller studies from Peru and Nepal also suggest a shorter reproductive lifespan in high altitude populations (50, 51). At a population level, observational studies in humans (35, 52) suggest that ovarian reserve reflects age at menopause, which is the best available proxy in women for the point at which unassisted conception becomes highly unlikely. Hence, our finding may translate into an important functional deficit in fertility, particularly in the older mother, following exposure to a suboptimal intrauterine environment. This is particularly relevant in many populations where age at first pregnancy is progressively increasing. A key advantage of the model used in this study (13% oxygen) is that it closely reflects the oxygenation during human pregnancy at altitude. At altitudes of 3000–3500 m above sea level, maternal arterial oxygen tension can fall to around 60% of the value at sea level [95 mmHg at sea level *vs.* 50 mmHg (26)]. The severity of the hypoxia used in our study is approximately equivalent to women experiencing pregnancy in the city of La Paz in Bolivia (3600–4150 m), where ∼40,000 women give birth annually (53). When considering high altitude populations, it is important to consider population mobility and thus the impact not only of prenatal hypoxia but also the postnatal environment on ovarian reserve into adulthood. This is an important area for future study.

As immediate survival of high-risk pregnancies improves (54, 55), it becomes increasingly important to understand the multitude of ways in which the health of survivors of adverse intrauterine environments may be affected in the longer term (56–58). Our study provides important novel evidence that fertility issues may also be among these programmed complications. Advances in assisted reproductive technologies mean that fertility problems are now often amenable to treatment, but this is much more likely to be successful if high-risk groups can be identified early in reproductive life (59). The finding that chronic fetal hypoxia results in decreased ovarian reserve in adulthood is therefore an important conceptual advance in understanding which future potential mothers are at high risk of experiencing fertility problems in later life. Moreover, our results provide mechanistic insight into how hypoxia-induced low ovarian reserve is associated with a specific defect in DNA repair and telomere maintenance in the somatic ovarian tissue. Insight into such molecular pathways is the first step toward developing effective interventions to protect ovarian reserve in the female offspring of high-risk pregnancy.

## Supplementary Material

This article includes supplemental data. Please visit *http://www.fasebj.org* to obtain this information.

## Abbreviations

Alox12: arachidonate 12-lipoxygenase
Alox15: arachidonate 15-lipoxygenase
Chk1: checkpoint kinase 1
Chk2: checkpoint kinase 2
CuZnsod: copper/zinc superoxide dismutase
DNA-PK: DNA-activated protein kinase
Ecsod: extracellular superoxide dismutase
Gp91^phox^: NADPH oxidase 1
Gpx1: glutathione peroxidase 1
H&E: hematoxylin and eosin
Hif1α: hypoxia-inducible factor 1α
Hmox1: heme oxygenase 1
Nrf2: nuclear respiratory factor 2
Ogg1: 8-oxoguanine DNA glycosylase
P16^ink^: cyclin-dependent kinase inhibitor 2A
P21: cyclin-dependent kinase inhibitor 1
P22^phox^: human neutrophil cytochrome b light chain
P53: tumor protein 53
P67phox: neutrophil cytosolic factor 2
Pot1: protection of telomeres protein 1
Ppia: cyclophilin A
Xo: xanthine oxidase
