# Supplementary figures and images for: Chronic gestational hypoxia accelerates ovarian aging and lowers ovarian reserve in next-generation adult rats
Source: FASEB J. 2019 Mar 19;33(6):7758–66. doi: 10.1096/fj.201802772R (PMC6529349; doi:10.1096/fj.201802772R)

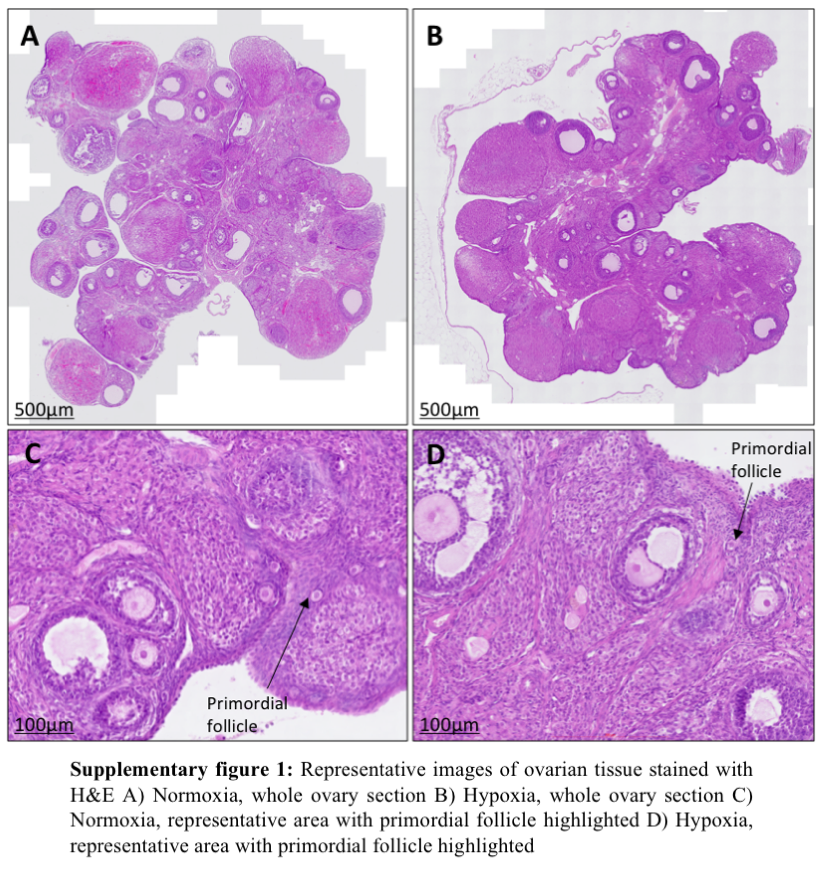

Supplement: Supplementary file 1 [file fj.201802772R.sf1.tif]

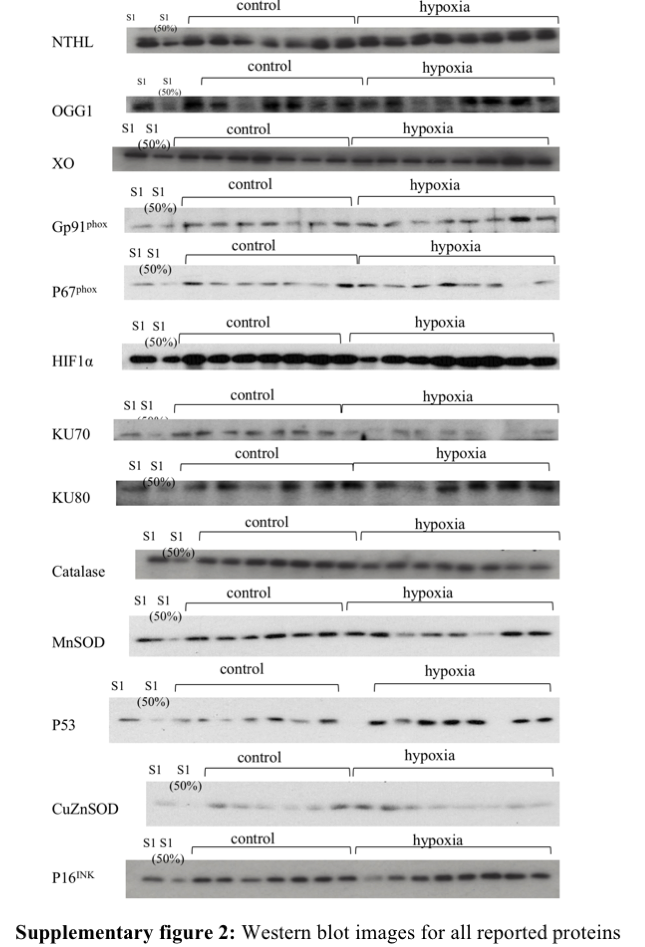

Supplement: Supplementary file 2 [file fj.201802772R.sf2.tif]
